# Non-surgical pain management for hip joint disease in veterinary medicine

**DOI:** 10.17221/19/2024-VETMED

**Published:** 2024-08-20

**Authors:** Jana Toholova, Slavomir Hornak, Maria Kuricova

**Affiliations:** Small Animal Clinic, University of Veterinary Medicine and Pharmacy, Košice, Slovak Republic

**Keywords:** hip dysplasia, mesenchymal stem cells, platelet-rich plasma, regenerative medicine

## Abstract

The most common orthopaedic developmental disease in dogs is hip dysplasia. This condition results in coxofemoral laxity due to incongruity and lack of stabilisation of the joint by the soft tissues. Currently, there is no therapeutic plan to correct hip dysplasia without surgical intervention at a very early age. The goal of the non-surgical treatment is to relieve pain and stiffness and to increase the muscle strength, usually through hydrotherapy and the beneficial physical properties of water. Recently, there has been growing interest in regenerative medicine, which involves the use of mesenchymal stem cells (MSCs) and their products to alleviate the characteristic clinical symptoms of osteoarthritis (OA). *In vivo* studies with canine MSCs have shown that an intra-articular injection of MSCs into cartilage lesions leads to the excellent regeneration of the hyaline cartilage. Regenerative medicine has undergone rapid development in recent years thanks to new therapies based on the application and combination of innovative biomaterials. One of the first known regenerative methods to be used in clinical practice was platelet-rich plasma (PRP). This review summarises the use and potential of MSCs and PRP, including their *in vitro* properties, their therapeutic effects in the treatment of cartilage lesions in preclinical *in vivo* studies, their clinical efficacy in the treatment of naturally occurring OA in dogs, and the current limitations of the studies.

## INTRODUCTION

**Figure 1:**
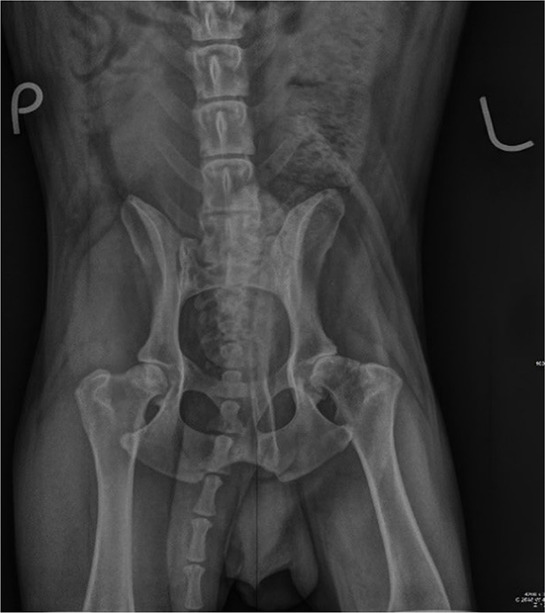
Radiographic findings of hip dysplasia in a dog

Hip dysplasia (HD) is an inherited developmental disorder of the hip joint that manifests itself as an abnormal structure of the acetabulum or the femoral head, or both (Henry 1992). The triggering factor for the development of dysplasia is the initial instability of the hip joint (Bardens and Hardwick 1968). The congruence of the articular surfaces of the hip joint is ensured by the joint capsule, the periarticular muscles and the ligaments of the femoral head, which is formed by the synovial fluid under conditions of mutual congruence of the articular surfaces. In canine hip dysplasia (CHD), the subluxation of the femoral head occurs most frequently leading to the destruction of the edges of the joint cavity and the flattening of the femoral head (Figure 1). There are many theories that explain the joint wear and tear in CHD, namely joint laxity and irregular endochondral ossification. These conditions are not mutually exclusive and their phenotypic expression is variable within the species. With a partially ossified hip structure, the hip joint may deform during development due to the mechanical stresses and the joint components may be more susceptible to deformation due to abnormal joint kinetics (Fujiki et al. 2007).

Abnormal endochondral ossification in the coxofemoral joint has been reported in 15-day-old dogs that developed hip dysplasia as early as at 12 months of age (Madsen et al. 1991) and also in Great Danes with experimentally induced hip dysplasia (Wu et al. 1974). In contrast, in Greyhounds, a breed in which dysplasia is among the least common, ossification of the joints appears to occur relatively early. Although it is clear that changes in the process of endochondral ossification may play a role in the development of hip dysplasia, the relationship between ossification patterns and the development of osteoarthritis remains unclear (Chalmers et al. 2006).

Recently, the dorsal acetabular rim angle (a measure of the dorsal slope of the subchondral bone surface in the acetabulum relative to the horizontal plane) was found to be significantly greater in dogs with coxofemoral laxity compared to normal dogs as early as at 1 week of age (Fujiki et al. 2007). The delayed ossification of the craniodorsal edge of the acetabulum is frequently observed at 8 weeks of age. Degeneration and micro-fractures of the articular cartilage as well as damage to the joint capsule, tendon insertions, and ligaments are often evident by 5 months of age (Barr et al. 1987). Hip laxity is considered a significant risk factor leading to abnormal forces and the subsequent development of osteoarthritis in adulthood (Ginja et al. 2009; Ginja et al. 2010). In general, there are two age groups at which animals show obvious clinical signs. The first group includes dogs younger than 1 year of age with hip instability, where the pain is mainly caused by synovitis and micro-fractures of the acetabulum (Riser 1975; Manley et al. 2007). The second group includes adult dogs with chronic pain due to osteoarthritis (Manley et al. 2007).

Typical clinical signs include gait disturbances such as reduced step height, stiffness and shortened stride length (Ginja et al. 2009). Evaluation of the hip joint is performed by orthopaedic radiographic imaging studies usually followed by preventive surgery if increased laxity is present, which occurs in most cases in puppies. The choice between conservative and surgical treatment depends on the age of the patient and the severity of the condition. The conservative treatment includes restriction of movement, body weight reduction and pain medication. Pain management strategies include the use of non-steroidal anti-inflammatory drugs (meloxicam, carprofen, derakoxib) or other adjunctive analgesics such as gabapentin, carprofen and corticosteroids (Pye et al. 2022). It focuses mainly on pain relief and alleviating the degenerative changes (Schachner and Lopez 2015). Other drugs, such as chondroprotectants, not only inhibit inflammatory mediators in the joint, but can also stimulate the metabolic activity of synoviocytes and chondrocytes. These drugs include glycosaminoglycan polysulfate esters, pentosan polysulphate and hyaluronic acid. Symptomatic medications are most commonly used to treat the hip pain and osteoarthritis (OA), to relieve pain or inflammation. However, their effect is relatively short-lived, only lasts for a certain period of time and can have various side effects such as the development of gastric ulcers. There are several joint supplements (such as chondroitin sulfate and glucosamine, polyunsaturated fatty acids, undenatured type I collagen), despite their great popularity, the efficacy and support from scientific studies in patients with degenerative joint disease is unclear (Guo et al. 2022). Methylsulfonylmethane (MSM) is an organic sulphur compound that occurs naturally and is one of the substances used in complementary and alternative medicine.

The authors Usha and Naidu (2004) investigated the effect of MSM in human medicine on knee osteoarthritis for 12 weeks in a supplementation group and a placebo group. The supplementation group showed a more significant reduction in pain. Methylensulfonylmethane and glucosamine were combined in the study. The result was a significant difference in the swelling index, joint mobility index, joint function and general functioning in favour of the supplemented group compared to the placebo group. Chondroprotectants include the polysulphated glycosaminoglycans (GAGPS), which also have anticoagulant and fibrinolytic properties (Ghosh et al. 1993). Although most of the scientific studies support the assumption that GAGPS have chondroprotective and chondrostimulating properties, some studies have found that GAGPS have no positive effect on the cartilage metabolism. In a clinical study of dogs with hip dysplasia, the greatest improvement in orthopaedic scores was seen at a dose of 4.4 mg/kg of Adequan administered intramuscularly over eight injections every 3 to 5 days (de Haan et al. 1994).

Another study found that an intramuscular administration of a dose of 5.0 mg/kg of GAGPS twice-weekly at 6 weeks to 8 months of age resulted in a lower incidence of coxofemoral subluxation in the growing puppies prone to hip dysplasia (Lust et al. 1992). The exact longevity of the relief achieved by GAGPS is not known.

Eckert et al. (2021) administered hydrolysed collagen to a dog with osteoarthritis over 16 weeks. The treatment resulted in increase in muscle and limb circumference and a significant reduction in pain symptoms.

Nutraceuticals and other dietary supplements have also become important tools in the treatment of OA in dogs and cats. These supplements appear to have the best effect in patients with mild or moderate OA. Mild OA can be treated initially with nutritional supplements with a non-steroidal anti-inflammatory drug (NSAID) if needed. Moderate OA is more likely to require a concurrent treatment with an NSAID and a dietary supplement. Surgical treatment is performed when a high degree of OA is present. Procedures include the total hip replacement, acetabular denervation, and femoral head and neck osteotomy (Ginja et al. 2010; Bergh and Budsberg 2014; Schachner and Lopez 2015). There are several objective limitations, such as the body weight of dogs undergoing a femoral head and neck ostectomy (Olsen et al. 2019).

Barlow, Ortolani and Barden tests are orthopaedic screening tests for the early diagnosis of dogs with possible hip dysplasia. The mechanism of these qualitative tests focuses on hip instability/laxity. All these tests can detect hip dysplasia. The Barlow test involves the dislocation of the femoral head, which is manually induced by a physician (Barlow 1962). The Ortolani test involves the retraction of the femoral head back into the acetabulum during luxation or subluxation of the hip joint (Chalman and Butler 1985). The Barden test is based on the horizontal-lateral mobility of the greater trochanter in relation to the tuber sacrale and tuber ischiadicum (Bardens and Hardwick 1968).

Goniometric measurements consist of measuring the reduction angle (RA) and the subluxation angle (SA). RA is the degree of femoral abduction at which the femoral head can be reduced or placed back into the acetabulum. The presence of RA is an indicator of expansion or dilation of the joint capsule. An RA in the range of 10° to 25° is considered mild joint laxity and an RA ≥ 25° is considered a high degree of joint laxity (Chalman and Butler 1985; Vezzoni et al. 2005). Hip dysplasia causes chronic pain and is currently an incurable and progressive disease in adult dogs. Due to the poor healing properties of cartilage lesions and the disability caused by chronic pain, the development of new therapies is a challenge. Regenerative medicine with stem cells and their products has the potential to overcome the associated limitations and improve the quality of life of affected dogs with new available methods of cartilage regeneration (Wang et al. 2017).

Recently, there has been growing interest in regenerative medicine, which involves the use of mesenchymal stem cells. These cells can differentiate into a variety of specific cells, including cartilage cells through the process of chondrogenesis (Abbaszadeh et al. 2020).

## CELL-FREE THERAPY

Due to its paracrine and immunomodulatory abilities, cell-free therapy is considered the ideal therapy for the treatment of inflammatory diseases of the musculoskeletal system (Ivanovska et al. 2022). For the treatment of osteoarthritis in dogs, cell-free therapy is commonly administered intravenously or as an intra-articular injection (Kriston-Pal et al. 2017; Olsen et al. 2019). Cell-free therapeutic strategies, such as artificial media and extracellular vesicles (EVs), could represent a possible perspective for MSC-based therapies in the treatment of osteoarthritis in dogs (Tofino-Vian et al. 2018).

The MSC-based treatment method has many advantages, it has a considerable effect in tumour therapy and pulmonary embolisation (Kang and Park 2014). In addition, problems such as the transmission of infections and difficulties with the low survival of MSC-transplanted cells are eliminated. Cell-free MSC-based technologies involve the use of modified conditioned media or membrane-bound extracellular vesicles (Sharun et al. 2022). MSC-derived conditioned media contain several biomolecules, such as chemokines, cytokines, and growth factors, that accelerate the regeneration of damaged tissue. In addition, MSCs also produce membrane-bound vesicles, including exosomes and microvesicles, which contribute to the therapeutic potential of the conditioned media (Hunakova et al. 2020; Sharun et al. 2022). Extracellular vesicles (EVs) are generally categorised by their size into microvesicles, exosomes, and apoptotic bodies (da Costa et al. 2021).

Exosomes and microvesicles play a key role in ensuring intercellular communication and apoptotic bodies being formed at apoptosis during cell division (Chandra and Sharma 2021; da Costa et al. 2021). Adipose tissue-derived MSC exosomes increased the expression of the anti-inflammatory cytokine IL-10 and decreased pro-inflammatory markers such as IL-6, tumour necrosis factor-α (TNF-α), and nuclear factor kappa B (NF-kB). In addition, the expression of anti-inflammatory cytokines increased when cultured with activated synovial fibroblasts. Furthermore, their effect on promoting chondrogenesis was confirmed by the increased abundance of markers such as β-catenin and type II collagen (Zhao et al. 2020).

Intra-articular injection of bone marrow-derived MSC microvesicles helped to restore the morphology of the damaged cartilage (Sabry et al. 2018). The conditioned medium obtained by isolation of adipose tissue-derived MSCs can be used to treat bilateral elbow joint osteoarthritis. The intra-articular injection of allogenic conditioned medium improved the locomotor abilities of Labrador Retriever dogs with osteoarthritis of the elbow joints (Hunakova et al. 2020).

MSC-derived exosomes slowed the development of osteoarthritis by inhibiting chondrocyte apoptosis and promoting extracellular matrix secretion (Tao et al. 2017). MSC-derived exosomes do not have the disadvantages of cell therapy (Joseph et al. 2020; Sharun et al. 2022). In addition, these MSC-derived exosomes contain many proteins, lipids, and ribonucleic acid that modulate homeostasis and regeneration (Zeng et al. 2022).

The extent of cartilage damage, osteophyte formation and subchondral sclerosis were significantly reduced in MSC-treated joints (Mokbel et al. 2011; Whitworth and Banks 2014). Kay et al. (2017) evaluated the therapeutic effect of conditioned medium from stem cells (CM-MSCs) as an alternative to cell-based therapy in an OA model. Cell-free therapeutic strategies, such as conditioned medium and EVs (exosomes), could potentially be the future of MSC-based therapies in the treatment of canine osteoarthritis (Mokbel et al. 2011; Whitworth and Banks 2014).

Vilar et al. (2013) demonstrated, in a canine model of OA, that an intra-articular injection of AD-MSCs (from allogenic canine adipose tissue derived MSCs) led to an improvement in limb function. Black et al. (2007) reported that an intra-articular injection of AD-MSCs led to better orthopaedic tests results. Several studies have investigated the mechanism of function of the conditioned medium (CM) in OA therapy. The CM was also harvested and used to isolate microvesicles and exosomes. These extracellular vesicles and the CM were used to treat chondrocytes from OA patients *in vitro*. The results showed that the CM, microvesicles and exosomes decreased the production of inflammatory mediators induced by IL1β, TNF-α and IL-6. At the same time, they reduced the production of PGE-2 and NO. Furthermore, the CM was able to reduce the IL1β-induced inflammatory effects and decrease the expression levels of thrombospondin motif metalloproteinase 5 (Simental-Mendia et al. 2020).

## HYDROTHERAPY

Physiotherapy is the therapeutic application of physical substances and stimuli, such as pressure, temperature, water, or movement to stimulate bodily functions (Samoy et al. 2018). In general, it aims to relieve pain and increase a joint’s range of motion (Prydie and Hewitt 2015a). Prydie and Hewitt (2015b) divided physiotherapy into two therapeutic groups, namely manual therapies and therapeutic exercises.

Indications for hydrotherapy, which is a therapeutic exercise, include post-operative rehabilitation, neurological conditions and the treatment of long-term pain (McGowan and Goff 2016a). The purpose of hydrotherapy is to increase the joint mobility (Sharp 2008; McGowan and Goff 2016a). Hydrotherapy can be used for prevention and treatment and is effective for maintaining overall fitness (Wong 2011).

The hydrostatic pressure expresses the pressure that the water exerts equally on all parts of the body in relation to the immersion depth of the body in the water. The deeper the body is immersed in the water, the higher the hydrostatic pressure (Wong 2011). The hydrostatic pressure exerts additional pressure on the chest, which improves the physical fitness by stimulation of the respiratory muscles (McGowan and Goff 2016b).

It is recommended that the temperature of the water is between 28 °C and 32 °C. Hot water releases the muscles and contributes to one’s well-being by relieving pain in the affected joints (Prydie and Hewitt 2015a). If the water is too cold, the blood vessels constrict, contributing to stiffness and discomfort. It is important to continuously monitor the patient during hydrotherapy as hyperthermia is a potential risk, especially in brachycephalic and obese dogs (Zink and Van Dyke 2013; McGowan and Goff 2016b). The primary goal of the non-surgical treatment of OA is to control pain and prevent any further progression of joint diseases (Dycus et al. 2017).

**Figure 2:**
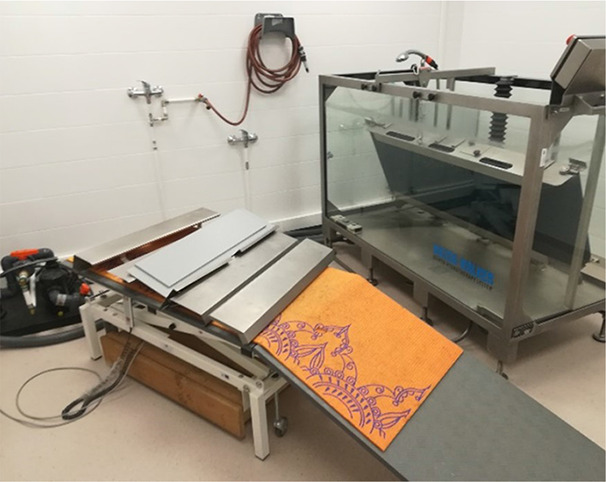
Underwater treadmill used for rehabilitation

Hydrotherapy primarily affects the muscles, ligaments, cartilage and the joint capsule (Millis and Levine 2014). The aim of hydrotherapy is to achieve an effect on all types of tissue. It is also used to retrain the gait and posture and to improve proprioception (Figure 2). Hydrotherapy also has a positive effect on cardiovascular and respiratory function. This therapeutic exercise can increase the flexibility of individual joints during swimming or underwater walking (Robertson 2003). Low-impact exercises, such as hydrotherapy, can prevent the exacerbation of osteoarthritis. Education and owner awareness of the need to control the body weight are the most important things to prevent osteoarthritis in dogs (Robertson 2003; Bland et al. 2009; Bland 2015). Obesity is a risk factor for the development of hip dysplasia and degenerative diseases in general (Kirkby and Lewis 2012). As long as dogs have a normal body condition score (BCS), their joints are less stressed compared to obese dogs.

According to Bland et al. (2009), 32% of dogs with proven hip dysplasia have a BCS of 4 or 5 out of 5 and are classified as obese. Hydrotherapy is therefore very important to promote the health and quality of life of dogs with hip joint diseases through a combination of body weight reduction and increased mobility of the affected joints.

## PLATELET-RICH PLASMA (PRP)

**Figure 3:**
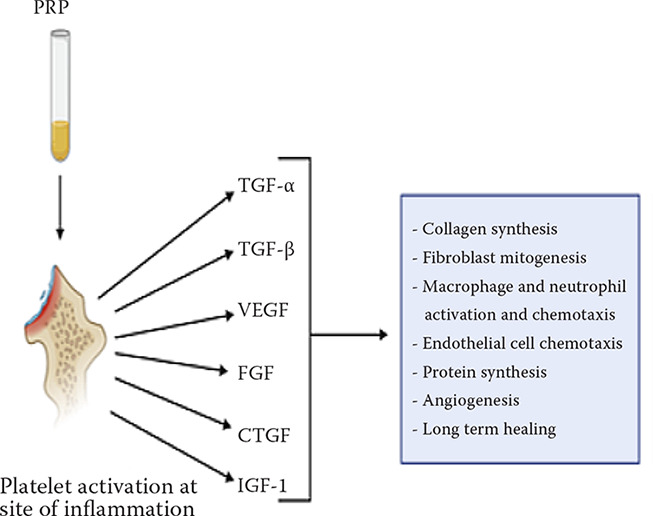
Platelet-rich plasma and specific growth factors (Hussain et al. 2017)

Platelet-rich plasma (PRP) was first used in the field of regenerative medicine in the 1970s. PRP was initially used to treat patients with thrombocytopaenia by PRP transfusion. Only later did it spread to the field of cardiovascular surgery, gynaecology and, above all, orthopaedic practice due to its anti-inflammatory and proliferative properties (Alves and Grimalt 2018). PRP is a product obtained from the patient’s own blood with a high blood content. Autologous PRP is produced by centrifuging the patient’s venous blood. Centrifugation separates and removes the platelets in the sample and buffy coat (erythrocytes and leukocytes) from the venous blood sample. The subsequent increased concentration of platelets in the gained sample promotes the healing of tissues damaged by traumatic and destructive conditions (Eppley et al. 2006). These platelets release growth factors and help to repair damaged tissue structures and periarticular soft tissues. In particular, these factors include specific platelet-derived growth factors (Figure 3) and the fibroblast growth factor (FGF), vascular endothelial growth factor (VEGF), transforming growth factors (TGF-α and TGF-β), connective tissue growth factor (CTGF) and insulin-like growth factor (IGF-1) (Hussain et al. 2017). Due to these biological properties, PRP therapy is suitable for therapeutic use in several medical fields, particularly in surgery and orthopaedics. Studies have shown that patients with hip osteoarthritis, elbow osteoarthritis and lateral epicondylitis can benefit from PRP therapy (Frechette et al. 2005).

The intra-articular application of platelet-rich plasma (PRP) can reduce or slow the progression of osteoarthritis by stimulating cartilage anabolism (Stief et al. 2011).

As PRP is an autologous blood product, there is no risk of disease transmission or immunological reaction. However, there may be redness at the injection site, swelling or even pain in the application area (Sampson et al. 2008). Sampson et al. (2010) reported significant pain relief and joint repair at the medial and lateral femoral condyle after three applications of PRP over a two-month period. Milano et al. (2010) investigated the effect of an intra-articular application of PRP in sheep with articular cartilage damage and found significant restoration of tissue damage compared to a control group by histologic evaluation. The most important advantages of autologous PRP include the absence of significant adverse effects or the transmission of infectious diseases. The intra-articular injection of PRP has been reported to have effects not only on articular cartilage, but also on other joint structures.

Moreover, PRP plays a complex role in inflammatory mechanisms and immunological responses. In addition to all these mechanisms, PRP also has a direct buffering effect (Lee et al. 2016). All these facts make it an important candidate for topical applications in orthopaedic conditions. Platelet-rich plasma shows promising and significant therapeutic potential in the treatment of hip osteoarthritis in large-breed dogs (Bland 2015). According to Bland (2015), adult to geriatric large breed dogs (Labrador Retrievers, German Shepherd dogs and German Pointing dogs) were predisposed to osteoarthritis (Bland 2015). In some dogs, there were no signs of pain or lameness in the treated joints. The allogenic administration of PRP has repeatedly been shown to be a rapid, effective and safe treatment for this condition.

## STEM CELL-BASED THERAPY

Mesenchymal stem cells (MSCs) are derived from the embryonic connective tissue or mesenchyme. Mesenchymal stem cells can differentiate into various tissues and have anti-inflammatory, immunomodulatory and immunosuppressive effects. They have the potential to preserve the articular cartilage and improve the joint function, which can subsequently relieve pain (Whitworth and Banks 2014). In adults, MSCs are found in connective tissues. Mesenchymal stem cells are commonly derived from bone marrow and adipose tissue, most commonly following an ovariohysterectomy (Beier et al. 2011). Adult MSCs have the ability to differentiate into specific cells, most commonly osteoblasts, adipocytes, and chondroblasts. They have the ability to self-renew depending on the environment (Gimble et al. 2008).

However, studies have shown that mesenchymal stem cells can target both ectodermal and endodermal cells *in vitro*. Examples of ectodermal cells are astrocytes and epithelial cells. MSCs have an immunomodulatory function that allows them to participate in cell therapy, especially with local or systemic inflammation. According to the available evidence, the intra-articular injection of mesenchymal stem cells is beneficial in the treatment of osteoarthritis in small animals (Kriston-Pal et al. 2017; Olsen et al. 2019). Mesenchymal stem cells in small animal practice can be isolated from various tissues such as the umbilical cord, muscle, synovial fluid or bone marrow (Sasaki et al. 2019). These stem cells can improve cartilage damage by secreting trophic and immunomodulatory factors, recruiting cells to the site of damage and redirecting them to differentiated cartilage tissues. (Sasaki et al. 2019; Domaniza et al. 2021).

MSCs are characterised by their differentiation potential and clonogenic capacity. According to the definition of the International Society for Cell Therapy, MSCs are characterised based on their three capacities, mainly their ability to adhere to plastic, their positive phenotype to release CD73, CD90, and CD105, their negative phenotype to release the CD features CD45, CD34 and CD14, and their differentiation into adipocytes, osteoblasts and chondroblasts (Dominici et al. 2006). Recent studies have shown the efficacy and usability of stem cells in the treatment of orthopaedic conditions, focusing on osteoarthritis in dogs. Maki et al. (2020) reported that, after treatment with stem cells, most dogs with hip osteoarthritis showed an improvement in lameness and an increase in serum interleukin 10 (Maki et al. 2020). Zhang et al. (2018) used umbilical cord-derived MSCs in small animal models of osteoarthritis and found a significant reduction in the blood levels of IL-6, IL-7 and TNF-α in the treatment group compared to the control group, suggesting a reduction in inflammation (Zhang et al. 2018). In another study, dogs with hip osteoarthritis received a single intra-articular injection of adipose-derived stem cells into the hip joint. A more successful outcome was reported than in the control patients and in patients injected with growth factor-rich plasma (Cuervo et al. 2014). *In vivo* studies with MSCs involved the use of mouse models of partial and full-thickness cartilage defects.

In each mouse model, stem cells were injected intra-articularly and showed significant regenerative effects compared to the control group (Miki et al. 2015).

The intra-articular application of MSCs often requires local or general anaesthesia. In addition, this procedure can be time-consuming in dogs with multiple OA (Olsen et al. 2019). Furthermore, *ex vivo* experiments have confirmed that osteoarthritic synovial fluid is cytotoxic to cultured MSCs (Kiefer et al. 2015). Therefore, the transplantation of viable stem cells into osteoarthritic joints should be considered a counter-productive procedure, as it reduces cell viability to the lowest possible level. In this sense, studies have confirmed that MSCs transplanted by intra-articular injection do not grow into the body’s own cartilage to effect direct repair (Desando et al. 2013; Satue et al. 2019). After the intra-articular injection of labelled adipose tissue-derived MSCs into the stifle joints of rabbits with osteoarthritis, MSCs were detected only in the medial meniscus and the synovial membrane, but not in the cartilage (Desando et al. 2013).

Virtually all research in the field of veterinary medicine has focused on adult stem cells, particularly bone marrow-derived MSCs (BM-MSCs) or adipose tissue-derived MSCs (AD-MSCs). The authors describe that BM-MSCs injections induce the regeneration of the hyaline cartilage and that the therapeutic effect of a delayed injection is limited.

Similar results were obtained for AD-MSCs. These results suggest that damaged tissue may express specific receptors or ligands that promote adhesion. Mesenchymal stem cells that pass into the synovium retain their properties for at least 28 days without transforming into another lineage. At the same time, they secrete several trophic factors such as PRG-4, BMP and TSG-6, which are the main trophic factors for chondroprotective and immunosuppressive function, which is an essential feature in the treatment of hip OA. Adipose tissue can be the preferred source of MSCs in small animals for several reasons: high productivity of MSCs (especially falciform fat), low morbidity, including ease of access and no additional pain associated with tissue harvesting (Ozeki et al. 2016).

Recent publications show that the intravenous application of stem cells does not allow direct access to joints or tissues, this method of application is currently not recommended in the field of orthopaedics (Harting et al. 2009). The authors conducted a study in 21 dogs with chronic hip OA (at least more than 6 months) (Black et al. 2007). Dogs treated with the intra-articular administration of autologous MSCs showed a significant decrease in pain, lameness scores and showed a higher range of motion of the joints compared to the control group (Black et al. 2007).

Another study (Cuervo et al. 2014) compared the efficacy of autologous MSCs versus PRP in the treatment of hip osteoarthritis. Thirty-nine dogs with hip OA were enrolled in this study and divided into two groups: 19 dogs received intra-articular injection of MSCs and 20 dogs received intra-articular injection of PRP. The results demonstrated that MSCs and PRP were a safe and effective treatment modality, as they significantly reduced pain and improved the physical activity 1, 3, and 6 months after treatment. Better long-term results were achieved in the MSC group at 6 months after treatment (Cuervo et al. 2014).

Vilar et al. (2013) recorded that the positive effect when combining MSCs with PRP was prolonged for more than 6 months. Furthermore, they found that dogs treated exclusively with MSCs showed more significant improvement over the first month after treatment, with decreased pain and lameness scores. However, this effect gradually diminished over 1 to 3 months (Vilar et al. 2014). In 2016, these authors compared the pain rating scales using force measurement platforms in the same animals over a 6-month period after application of MSCs. This research was conducted using a pain rating scale to measure OA-related lameness; however, it did not show accuracy when compared to a quantitative kinematic analysis (Vilar et al. 2016).

## CONCLUSION

Recently, there has been increased interest in regenerative medicine and many studies have tried to demonstrate the increasing potential of MSCs. Their use in new therapeutic approaches seems inevitable. The basic properties of MSCs include the ability to proliferate without a loss of characteristics and the ability to differentiate into specific types of cells. In addition, their properties include immunomodulatory functions, the ability to release trophic and immunomodulatory factors and to influence their environment at the same time. The development of knowledge in the field of regenerative medicine will allow the further development of new therapeutic strategies. There are many factors regarding the most appropriate route of the MSC application, optimal source of tissue and impact of stem cells on the donor’s condition, which still need to be more deeply investigated and reconsidered. For this reason, when selecting a donor, screening for infectious diseases and other risky diseases and factors that are important for the safety of the animal involved in the study is carried out.

It is therefore essential to know the origin and the composition of the product and the storage conditions of the product. It is necessary to demonstrate the functionality and integrity of the cells during the study process and to prove that the stem cells are not contaminated by viruses and bacteria.

In conclusion, platelet-rich plasma also shows a significant therapeutic potential especially in the field of surgery and orthopaedics, in the treatment of osteoarthritis in dogs. Regenerative medicine is of great use in preventing acute osteoarthritis changes in the elbow and hip joints, improving the mobility of the joint and relieving pain during movements. Further investigations and prospective clinical studies are needed to validate the findings in the field of small animal practice in order to make conclusive statements.

## References

[R1] Abbaszadeh H, Ghorbani F, Derakhshani M, Movassaghpour AAA, Yousefi M, Talebi M, Shamsasenjan K. Regenerative potential of Wharton’s jelly-derived mesenchymal stem cells: A new horizon of stem cell therapy. J Cell Physiol. 2020 Dec;235(12):9230-40. 32557631 10.1002/jcp.29810

[R2] Alves R, Grimalt R. A review of platelet-rich plasma: History, biology, mechanism of action, and classification. Skin Appendage Disord. 2018 Jan;4(1):18-24. 29457008 10.1159/000477353 PMC5806188

[R3] Bardens JW, Hardwick H. New observations on the diagnosis and cause of hip dysplasia. Vet Med Small Anim Clin. 1968 Mar;63(3):238-45. 5183841

[R4] Barlow TG. Early diagnosis and treatment of congenital dislocation of the hip. J Bone Joint Surg Br. 1962;44(2):292-301.

[R5] Barr ARS, Denny HR, Gibbs C. Clinical hip dysplasia in growing dogs: The long-term results of conservative management. J Small Anim Pract. 1987 Apr 1;28(4):243-52.

[R6] Beier JP, Bitto FF, Lange C, Klumpp D, Arkudas A, Bleiziffer O, Boos AM, Horch RE, Kneser U. Myogenic differentiation of mesenchymal stem cells co-cultured with primary myoblasts. Cell Biol Int. 2011 Apr;35(4):397-406. 20946104 10.1042/CBI20100417

[R7] Bergh MS, Budsberg SC. A systematic review of the literature describing the efficacy of surgical treatments for canine hip dysplasia. Vet Surg. 2014 Jul;43(5):501-6. 24837650 10.1111/j.1532-950X.2014.12208.x

[R8] Black LL, Gaynor J, Gahring D, Adams C, Aron D, Harman S, Gingerich DA. Effect of adipose-derived mesenchymal stem and regenerative cells on lameness in dogs with chronic osteoarthritis of the coxofemoral joints: A randomized, double-blinded, multicenter, controlled trial. Vet Ther. 2007;8(4):272-84. 18183546

[R9] Bland SD. Canine osteoarthritis and treatments: A review. Vet Sci Dev. 2015;5(2):84-9.

[R10] Bland IM, Guthrie-Jones A, Taylor RD, Hill J. Dog obesity: Owner attitudes and behaviour. Prev Vet Med. 2009 Dec;92(4):333-40. 19766333 10.1016/j.prevetmed.2009.08.016

[R11] Chalman JA, Butler HC. Coxofemoral joint laxity and the Ortolani sign. J Am Anim Hosp Assoc. 1985;21(5):671-6.

[R12] Chalmers HJ, Dykes NL, Lust G, Farese JP, Burton-Wuster NI, Williams AJ, Todhunter RJ. Assessment of bone mineral density of the femoral head in dogs with early osteoarthritis. Am J Vet Res. 2006 May;67(5):796-800. 16649912 10.2460/ajvr.67.5.796

[R13] Chandra V, Sharma GT. Prospects of mesenchymal stem cell secretome in veterinary regenerative therapy. In: Choudhary RK, Choudhary S, editors. Stem cells in veterinary science. Singapore: Springer; 2021. p. 271-87.

[R14] Cuervo B, Rubio M, Sopena J, Dominguez JM, Vilar J, Morales M, Cugat R, Carrillo JM. Hip osteoarthritis in dogs: A randomized study using mesenchymal stem cells from adipose tissue and plasma rich in growth factors. Int J Mol Sci. 2014;15(8):13437-60. 25089877 10.3390/ijms150813437 PMC4159804

[R15] da Costa VR, Araldi RP, Vigerelli H, D’Amelio F, Mendes TB, Gonzaga V, Policiquio B, Colozza-Gama GA, Valverde CW, Kerkis I. Exosomes in the tumor microenvironment: From biology to clinical applications. Cell. 2021 Oct 1;10(10):2617. 10.3390/cells10102617 PMC8533895 34685596

[R16] de Haan JJ, Goring RL, Beale BS. Evaluation of polysulfated gllycosaminoglycan for the treatment of hip dysplasia in dogs. Vet Surg. 1994 May-Jun;23(3):177-81. 8066981 10.1111/j.1532-950x.1994.tb00468.x

[R17] Desando G, Cavallo C, Sartoni F, Martini L, Parrilli A, Veronesi F, Fini M, Giardino R, Facchini A, Grigolo B. Intra-articular delivery of adipose derived stromal cells attenuates osteoarthritis progression in an experimental rabbit model. Arthritis Res Ther. 2013 Jan 29;15(1):R22. 23360790 10.1186/ar4156 PMC3672720

[R18] Domaniza M, Trbolova A, Hluchy M, Cizkova D. Mesenchymal stem cell-based treatment of osteoarthritis in dogs – A review. J Stem Cell Res Dev Ther. 2021 Oct;7(4):17.

[R19] Dominici M, Le Blanc K, Mueller I, Slaper-Cortenbach I, Marini F, Krause D, Deans R, Keating A, Prockop D, Horwitz E. Minimal criteria for defining multipotent mesenchymal stromal cells. The International Society for Cellular Therapy position statement. Cytotherapy. 2006;8(4):315-7. 16923606 10.1080/14653240600855905

[R20] Dycus D, Levine D, Marcellin-Little D. Physical rehabilitation for the management of canine hip dysplasia. Vet Clin Small Anim. 2017 Jul;47(4):823-50. 10.1016/j.cvsm.2017.02.006 28576271

[R21] Eckert T, Jaehrling-Butkus M, Louton H, Burg-Roderfeld M, Zhang R, Zhang N, Siebert HC. Efficacy of chondroprotective food supplements based on collagen hydrolysate and compounds isolated from marine organisms. Mar Drugs. 2021 Sep 26;19(10):542. 34677442 10.3390/md19100542 PMC8541357

[R22] Eppley BL, Pietrzak WS, Blanton M. Platelet-rich plasma: A review of biology and applications in plastic surgery. Plast Reconstr Surg. 2006 Nov;118(6):147-59. 10.1097/01.prs.0000239606.92676.cf 17051095

[R23] Frechette JP, Martineau I, Gagnon G. Plateletrich plasmas: Growth factor content and roles in wound healing. J Dent Res. 2005 May;84(5):434-9. 15840779 10.1177/154405910508400507

[R24] Fujiki M, Kurima Y, Yamanokuchi K, Misumi K, Sakamoto H. Computed tomographic evaluation of growth-related changes in the hip joints of young dogs. Am J Vet Res. 2007 Jul;68(7):730-4. 17605608 10.2460/ajvr.68.7.730

[R25] Ghosh P, Smith M, Wells C. Second-line agents in osteoarthritis. In: Dixon JS, Furst DE, editors. Second-line agents in the treatment of rheumatic diseases. New York: Marcel Dekker; 1993. p. 363-427.

[R26] Gimble JM, Guilak F, Nuttall ME, Sathishkumar S, Vidal M. In vitro differentiation potential of mesenchymalstemcells. Transfus Med Hemother. 2008 Jun;35(3): 228-38. 21547120 10.1159/000124281 PMC3083290

[R27] Ginja MMD, Ferreira AJ, Jesus SS, Melo-Pinto P, Bulas-Cruz J, Orden MA, Gonzalo-Orden JM. Comparison of clinical, radiographic, computed tomographic and magnetic resonance imaging methods for early prediction of canine hip dysplasia. Vet Radiol Ultrasound. 2009 Mar-Apr;50(2):135-43. 19400458 10.1111/j.1740-8261.2009.01506.x

[R28] Ginja MMD, Silvestre AM, Gonzalo-Orden JM, Ferreira AJA. Diagnosis, genetic control and preventive management of canine hip dysplasia: A review. Vet J. 2010 Jun;84(3):269-76. 10.1016/j.tvjl.2009.04.009 19428274

[R29] Guo W, Douma L, Hu MH, Eglin D, Alini M, Secerovic A, Grad S, Peng X, Zou X, D’Este M, Peroglio M. Hyaluronic acid-based interpenetrating network hydrogel as a cell carrier for nucleus pulposus repair. Carbohydr Polym. 2022 Feb 1;277:118828. 34893245 10.1016/j.carbpol.2021.118828

[R30] Harting MT, Jimenez F, Xue H, Fischer UM, Baumgartner J, Dash PK, Cox CS. Intravenous mesenchymal stem cell therapy for traumatic brain injury. J Neurosurg. 2009 Jun;110(6):1189-97. 19301973 10.3171/2008.9.JNS08158 PMC2889620

[R31] Henry GA. Radiographic development of canine hip dysplasia. Vet Clin North Am Small Anim Pract. 1992 May;22(3):559-78. 1604773 10.1016/s0195-5616(92)50056-0

[R32] Hunakova K, Hluchy M, Spakova T, Matejova J, Mudronova D, Kuricova M, Rosocha J, Ledecky V. Study of bilateral lake joint osteoarthritis treatment using conditioning medium from allogeneic adipose tissue-derivedMSCs in Labrador Retrievers. Res Vet Sci. 2020 Oct 1;132:513-20. 32805699 10.1016/j.rvsc.2020.08.004

[R33] Hussain N, Johal H, Bhandari M. An evidence-based evaluation on the use of platelet rich plasma in orthopedics – A review of the literature. SICOT J. 2017;3:57. 28990574 10.1051/sicotj/2017036 PMC5632954

[R34] Ivanovska A, Wang M, Arshaghi TE, Shaw G, Alves J, Byrne A, Butterworth S, Chandler R, Cuddy L, Dunne J, Guerin S, Harry R, McAlindan A, Mullins RA, Barry F. Manufacturing mesenchymal stromal cells for thetreatment of osteoarthritis in canine patients: Challenges and recommendations. Front Vet Sci. 2022 Jun;9:897150. 35754551 10.3389/fvets.2022.897150 PMC9230578

[R35] Joseph A, Baiju I, Bhat IA, Pandey S, Bharti M, Verma M, Pratap Singh A, Ansari MM, Chandra V, Saikumar G, Amarpal A, Sharma GT. Mesenchymal stem cell-conditioned media: A novel alternative of stem cell therapy for quality wound healing. J Cell Physiol. 2020 Jan;235(12):5555-69. 31960454 10.1002/jcp.29486

[R36] Kang MH, Park HM. Evaluation of adverse reactions in dogs following intravenous mesenchymal stem cell transplantation. Acta Vet Scand. 2014 Mar;56(1):16. 24655411 10.1186/1751-0147-56-16 PMC3994522

[R37] Kay AG, Long G, Tyler G, Stefan A, Broadfoot SJ, Piccinini AM, Kehoe O. Mesenchymal stem cell-conditioned medium reduces disease severity and immune responses in inflammatory arthritis. Sci Rep. 2017;7(1):18019. 29269885 10.1038/s41598-017-18144-w PMC5740178

[R38] Kiefer KM, O’Brien TD, Pluhar EG, Conzemius M. Canine adipose-derived stromal cell viability following exposure to synovial fluid from osteoarthritic joints. Vet Rec Open. 2015 Jul;2(1):e000063. 26392889 10.1136/vetreco-2014-000063 PMC4567176

[R39] Kirkby KA, Lewis D. Canine hip dysplasia: Reviewing the evidence for nonsurgical management. Vet Surg. 2012 Jan;41(1):2-9. 22150604 10.1111/j.1532-950X.2011.00928.x

[R40] Kriston-Pal E, Czibula A, Gyuris Z, Balka G, Seregi A, Sukosd F, Suth M, Kiss-Toth E, Haracska L, Uher F, Monostori E. Characterization and therapeutic application of canine adipose mesenchymal stem cells to treat elbow osteo-arthritis. Can J Vet Res. 2017 Jan;81(1):73-8. 28197017 PMC5220603

[R41] Lee HR, Shon OJ, Park SI, Kim HJ, Kim S, Ahn MW, Do SH. Platelet-rich plasma increases the levels ofcatabolic molecules and cellular dedifferentiation in the meniscus of a rabbit model. Int J Mol Sci. 2016 Jan;17(1):120. 26784189 10.3390/ijms17010120 PMC4730361

[R42] Lust G, Williams AJ, Burton-Wurster N, Beck KA, Rubin G. Effects of intramuscular administration of glycosaminoglycan polysulfates on signs of incipient hip dysplasia in growing pups. Am J Vet Res, 1992 Oct;53(10):1836-43. 1456530

[R43] Madsen JS, Reimann I, Svalastoga E. Delayed ossification of the femoral head in dogs with hip dysplasia. J Small Anim Pract. 1991 Jul;32:351-4.

[R44] Maki CB, Beck A, Wallis C, Choo J, Raos T, Tong R, Borjesson DL, Izadyar F. Intra-articular administration of allogeneic adipose derived mscs reduces pain and lameness in dogs with hip osteoarthritis: A double blinded, randomized, placebo controlled pilot study. Front Vet Sci. 2020 Aug;7:570. 33110913 10.3389/fvets.2020.00570 PMC7489271

[R45] Manley PA, Adams WM, Danielson KC, Dueland RT, Linn KA. Long-term outcome of juvenile pubic symphysiodesis and triple pelvic osteotomy in dogs with hip dysplasia. J Am Vet Med Assoc. 2007 Jan;230(2):206-10. 17223752 10.2460/javma.230.2.206

[R46] McGowan C, Goff L. Canine therapy and rehabilitation for orthopaedic conditions. In: Goff S, Stubbs N, editors. Animal physiotherapy: Assessment, treatment and rehabilitation of animals. 2^nd^ ed. Chichester, West Sussex: Wiley-Blackwell; 2016a. p. 272-301.

[R47] McGowan C, Goff L. Aquatic theray. In: Goff S, Stubbs N, editors. Animal physiotherapy: Assessment, treatment and rehabilitation of animals. 2^nd^ ed. Chichester, West Sussex: Wiley-Blackwell; 2016b. p. 225-37.

[R48] Miki S, Takao M, Miyamoto W, Matsushita T, Kawano H. Intra-articular injection of mesenchymal stem cell-derived synovial hyaluronic acid can repair articular cartilage defects in a canine model. J Stem Cell Res Ther. 2015 Jan;5(11).

[R49] Milano G, Sanna Passino E, Deriu L, Careddu G, Manunta L, Manunta A, Saccomanno MF, Fabbriciani C. The effect of platelet rich plasma combined with microfractures on the treatment of chondral defects: An experimental study in a sheep model. Osteoarthritis Cartilage. 2010 Jul;18(7):971-80. 20433936 10.1016/j.joca.2010.03.013

[R50] Millis DL, Levine D. Canine rehabilitation and physical therapy. 2^nd^ ed. Philadelphia: Elsevier Health Sciences; 2014. 784 p.

[R51] Mokbel AN, El Tookhy OS, Shamaa AA, Rashed LA, Sabry D, El Sayed AM. Homing and reparative effect of intra-articular injection of autologus mesenchymal stem cells in osteoarthritic animal model. BMC Musculoskelet Disord. 2011 Nov 15;12:259. 22085445 10.1186/1471-2474-12-259 PMC3232438

[R52] Olsen A, Johnson V, Webb T, Santangelo KS, Dow S, Duerr FM. Evaluation of intravenously delivered allogeneic mesenchymal stem cells for treatment of elbow osteoarthritis in dogs: A pilot study. Vet Comp Orthop Traumatol. 2019 May;32(3):173-81. 30873568 10.1055/s-0039-1678547

[R53] Ozeki N, Muneta T, Koga H, Nakagawa Y, Mizuno M, Tsuji K, Mabuchi Y, Akazawa C, Kobayashi E, Matsumoto K, Futamura K, Saito T, Sekiya I. Not single but periodic injections of synovial mesenchymal stem cell maintain viable cells in knees and inhibit the progression of osteoarthritis in rats. Osteoarthritis Cartilage. 2016 Jun;24(6):1061-70. 26880531 10.1016/j.joca.2015.12.018

[R54] Prydie D, Hewitt I. Modalities. In: Prydie D, Hewitt I, editors. Practical physiotherapy for small animal practice. 1^st^ ed. West Sussex: Wiley-Blackwell; 2015a. p. 69-90.

[R55] Prydie D, Hewitt I. Treatment protocols for hip dysplasia. In: In: Prydie D, Hewitt I, editors. Practical physiotherapy for small animal practice. 1^st^ ed. West Sussex: Wiley-Blackwell; 2015b. p. 237-40.

[R56] Pye C, Bruniges N, Peffers M, Comerford E. Advances in the pharmaceutical treatment options for canine osteoarthritis. J Small Anim Pract. 2022 Mar;63(10):721-38. 35285032 10.1111/jsap.13495 PMC9790257

[R57] Riser WH. The dog as a model for the study of hip dysplasia: Growth, form, and development of normal and dysplastic hip joint. Vet Pathol. 1975;12(4):244-334. 10.1177/030098587501200401 1224531

[R58] Robertson ID. The association of exercise, diet and other factors with owner perceived obesity in privately owned dogs from metropolitan perth. Prev Vet Med. 2003 Apr;58(1-2):75-83. 12628772 10.1016/s0167-5877(03)00009-6

[R59] Sabry D, Shamaa A, Amer M, El-Tookhy O, Abdallah A, Abd El Hassib DM, Amer E, Elamir A. The effect of mesenchymal stem cell derived microvesicles in repair of femoral chondral defects in dogs. J Musculoskelet Res. 2018;21(2):1850006.

[R60] Samoy Y, Van Ryssen B, Saunders J. Physiotherapy in small animal medicine. Vlaams Diergeneeskundig Tijdschrift. 2018;85:323-34.

[R61] Sampson S, Gerhardt M, Mandelbaum B. Platelet rich plasma injection grafts for musculoskeletal injuries: A review. Curr Rev Musculoskelet Med. 2008 Dec;1(3-4):165-74. 19468902 10.1007/s12178-008-9032-5 PMC2682411

[R62] Sampson S, Reed M, Silvers H, Meng M, Mandelbaum B. Injection of platelet-rich plasma in patients with primary and secondary knee osteoarthritis: A pilot study. Am J Phys Med Rehabil. 2010 Dec;89(12):961-9. 21403592 10.1097/PHM.0b013e3181fc7edf

[R63] Sasaki A, Mizuno M, Mochizuki M, Sekiya I. Mesenchymal stem cells for cartilage regeneration in dogs. World J Stem Cells. 2019 May;11(5):254-69. 31171954 10.4252/wjsc.v11.i5.254 PMC6545524

[R64] Satue M, Schueler C, Ginner N, Erben RG. Intra-articularly injected mesenchymal stem cells promote cartilage regeneration, but do not permanently engraft in distant organs. Sci Rep. 2019 Jul;9(1):10153. 31300685 10.1038/s41598-019-46554-5 PMC6626061

[R65] Schachner ER, Lopez MJ. Diagnosis, prevention, and management of canine hip dysplasia: A review. Vet Med (Auckl). 2015 May 19;6:181-92. 30101105 10.2147/VMRR.S53266 PMC6070021

[R66] Sharp B. Physiotherapy in small animal practice. In Pract. 2008 Apr;30(4):190-9.

[R67] Sharun K, Dhama K, Jambagi K, Pawde AM. Cell-free therapy for inflammatory diseases: opportunities and challenges. Recent Adv Inflamm Allergy Drug Discov. 2022;15(1):5-8. 34931977 10.2174/2772270816666211220152218

[R68] Simental-Mendia M, Lozano-Sepulveda SA, Perez-Silos V, Fuentes-Mera L, Martinez-Rodriguez HG, Acosta-Olivo CA, Pena-Martinez VM, Vilchez-Cavazos F. Anti-inflammatory and anti-catabolic effect of non-animal stabilized hyaluronic acid and mesenchymal stem cell-conditioned medium in an osteoarthritis coculture model. Mol Med Rep. 2020 May;21(5):2243-50. 32323772 10.3892/mmr.2020.11004

[R69] Stief M, Gottschalk J, Ionita JC, Einspanier A, Oechtering G, Boettcher P. Concentration of platelets and growth factors in canine autologous conditioned plasma. Vet Comp Orthop Traumatol. 2011 Jan;24(2):122-5. 21225088 10.3415/VCOT-10-04-0064

[R70] Tao SC, Yuan T, Zhang YL, Yin WJ, Guo SC, Zhang CQ. Exosomes derived from miR-140-5p-overexpressing human synovial mesenchymal stem cells enhance cartilage tissue regeneration and prevent osteoarthritis of the knee in a rat model. Theranostics. 2017 Jan 1;7(1):180-95. 28042326 10.7150/thno.17133 PMC5196895

[R71] Tofino-Vian M, Guillen MI, Alcaraz MJ. Extracellular vesicles: A new therapeutic strategy for joint conditions. Biochem Pharmacol. 2018 Jul;153:134-46. 29427625 10.1016/j.bcp.2018.02.004

[R72] Usha PR, Naidu MUR. Randomized, double-blinded parallel, placebo-controlled study of oral glucosamin, methylsulfonylmethane and their combination in osteoartritis. Clin Drug Invest. 2004;24(6):353-63. 10.2165/00044011-200424060-00005 17516722

[R73] Vezzoni A, Dravelli G, Corbari A, De Lorenzi M, Cirla A, Tranquilo V. Early diagnosis of canine hip dysplasia. Eur J Comp Anim Pract. 2005;15:173-83.

[R74] Vilar JM, Batista M, Morales M, Santana A, Cuervo B, Rubio M, Carrillo JM. Assessment of the effect of intraarticular injection of autologous adipose-derived mesenchymal stem cells in osteoarthritic dogs using a double blinded force platform analysis. BMC Vet Res. 2014 Jul 1;10:143. 24984756 10.1186/1746-6148-10-143 PMC4085658

[R75] Vilar JM, Cuervo B, Rubio M, Sopena J, Dominguez JM, Santana A, Carrillo JM. Effect of intraarticular inoculation of mesenchymal stem cells in dogs with hip osteoarthritis by means of objective force platform gait analysis: Concordance with numeric subjective scoring scales. BMC Vet Res. 2016 Oct;12(1):223. 27717361 10.1186/s12917-016-0852-z PMC5055672

[R76] Vilar JM, Morales M, Santana A, Spinella G, Rubio M, Cuervo B, Cugat R, Carrillo JM. Controlled, blinded force platform analysis of the effect of intraarticular injection of autologous adipose-derived mesenchymal stem cells associated to PRGF-Endoret in osteoarthritic dogs. BMC Vet Res. 2013 Jul 2;9:131. 23819757 10.1186/1746-6148-9-131 PMC3716942

[R77] Wang H, Shan XB, Qiao YJ. PDK2 promotes chondrogenic differentiation of mesenchymal stem cells by upregulation of Sox6 and activation of JNK/MAPK/ERK pathway. Braz J Med Biol Res. 2017 Feb;50(2):e5988. 28225870 10.1590/1414-431X20165988 PMC5343558

[R78] Whitworth DJ, Banks TA. Stem cell therapies for treating osteoarthritis: Prescient or premature? Vet J. 2014 Dec;202(3):416-24. 25457267 10.1016/j.tvjl.2014.09.024

[R79] Wong E. Hip dysplasia. In: Wong E, editor. Swim to recovery: Canine hydrotherapy healing. 1^st^ ed. Dorchester, England: Hubble & Hattie; 2011. p. 55-69.

[R80] Wu FM, Hedhammar A, Krook L. Overnutrition and skeletal disease. An experimental study in growing Great Dane dogs. IX. The long bones. Cornell Vet. 1974 Apr;64(2):Suppl_5:83-114. 4826275

[R81] Zeng Z, Dai Y, Deng S, Zou S, Dou T, Wei F. Synovial mesenchymal stem cell-derived extracellular vesicles alleviate chondrocyte damage during osteoarthritis through microRNA-130b-3p-mediated inhibition of the LRP12/AKT/β-catenin axis. Immunopharmacol Immunotoxicol. 2022 Jan;44(2):247-60. 35174753 10.1080/08923973.2022.2038192

[R82] Zhang BY, Wang BY, Li SC, Luo DZ, Zhan X, Chen SF, He Y. Evaluation of the curative effect of umbilical cord mesenchymal stem cell therapy for knee arthritis in dogs using imaging technology. Stem Cells Int. 2018 May;1:1983025. 10.1155/2018/1983025 PMC5976915 29861739

[R83] Zhao C, Chen JY, Peng WM, Yuan B, Bi Q, Xu YJ. Exosomes from adipose-derived stem cells promote chondrogenesis and suppress inflammation by upregulating miR-145 and miR-221. Mol Med Rep. 2020 Apr;21(4):1881-9. 32319611 10.3892/mmr.2020.10982 PMC7057766

[R84] Zink C, Van Dyke JB. Canine sports medicine and rehabilitation. 1^st^ ed. Ames, Iowa: Wiley-Blackwell; 2013. p. 159-75.

